# Performance Evaluation of the Fully Automated NeuMoDx RT-PCR Platform for the Quantification of CMV and EBV DNA in EDTA Plasma: Implications for Clinical Management and Establishment of a Conversion Formula

**DOI:** 10.1128/spectrum.02157-22

**Published:** 2022-11-07

**Authors:** Anna Nele Herdina, Franz Ratzinger, Monika Breuer, Julia Schellnegger, Rui Qiang Chen, Thomas Watkins-Riedel, Nicole Perkmann-Nagele, Robert Strassl

**Affiliations:** a Division of Clinical Virology, Department of Laboratory Medicine, Medical University of Vienna, Vienna, Austria; b IhrLabor, Medical Diagnostic Laboratories, Vienna, Austria; Quest Diagnostics Nichols Institute

**Keywords:** cytomegalovirus, Epstein-Barr virus, solid organ transplant (SOT), reactivation, transplant management

## Abstract

The NeuMoDx96 platform is a fully automated real-time PCR (RT-PCR) system. To provide continued testing quality with the introduction of new assays, the primary aim of this study was to evaluate the analytical and clinical performance of the NeuMoDx platform for the detection and quantification of CMV and EBV DNA in EDTA plasma. As no conversion from log_10_ international units per milliliter to copies per milliliter was provided, the secondary aim was to calculate and establish a conversion factor for the output of results in copies per milliliter for CMV and EBV. Archived ETDA plasma samples (cytomegalovirus [CMV], *n* = 290; Ebstein-Barr virus [EBV], *n* = 254) were used to evaluate the analytical performance of the NeuMoDx96 platform against the routine real-time quantitative PCR (qPCR) assays. Additionally, the first WHO international standards (WHO-IS) for CMV (*n* = 70) and EBV (*n* = 72) were used for the calculation of the intra- and interassay variation. WHO-IS qualitative agreement between the assays was 100%. Intra-assay variability was low for both CMV assays (coefficient of variation [CV], phosphate-buffered saline [PBS], 3 log_10_ IU/mL NeuMoDx, 3.67%; Abbott RealTime, CMV, 3.35%) and NeuMoDx EBV assay (CV, PBS, 3 log_10_ IU/mL, 3.05%) but high for the Altona EBV assay (CV, PBS, 3 log_10_ IU/mL, 26.13%). The overall qualitative concordance in clinical samples was 96.8% (270/279) for CMV and 96.7% (237/245) for EBV. The mean difference between the assays was −0.2 log_10_ IU/mL (CMV) and −0.18 log_10_ IU/mL (EBV). High qualitative concordance and a significant correlation of quantitative values for both assays make NeuMoDx CMV and EBV assays suitable for routine diagnostic testing. The new RT-PCR system and conversion formulas to report results in copies per milliliter are now applied in clinical routine testing.

**IMPORTANCE** Clinical management of solid organ transplant (SOT) patients requires the careful monitoring of immunosuppression and viral infection or reactivation. qPCR is the gold standard for the detection and quantification of very small amounts of viral DNA and allows for an early assessment of viral load kinetics. The tested NeuMoDx 96 platform provides faster results than the previously used RT-PCR workflows for CMV (Abbott m2000 and RealTime CMV assay) and EBV (LightCycler 480 II, Roche high pure extraction, and Altona RealStar EBV assay) DNA detection. The implemented conversion formulas allow the continued reporting in clinically established copies per milliliter, important for long-term care of SOT patients.

## INTRODUCTION

Cytomegalovirus (CMV) and Epstein-Barr virus (EBV) belong to the family *Herpesviridae*, which comprises a group of 17 herpesvirus genera. Nine herpesvirus species (HHVs) typically cause infections only in humans. Morphologically, HHVs have a large double-stranded DNA genome and replicate in the nucleus of the host cell (1). HHVs are widespread all over the world, with seroprevalences in human populations ranging between 30% and 100%, depending on the virus, age group, geographical variations, and socioeconomic factors (2, 3). CMV belongs to the subfamily *Betaherpesvirinae* and is recognized as the most common congenital viral infection in humans, with an estimated global seroprevalence of 83% (95% uncertainty interval [UI], 78 to 88) (4). There are, however, regions with reported CMV seroprevalences in adults of up to 100% (5). EBV, which infects 90 to 95% of humans worldwide, is a gammaherpesvirus and is the causative agent of infectious mononucleosis (6). As with all HHVs, following primary infection, CMV and EBV characteristically establish a lifelong latent infection in the host, with the ability to reactivate. In healthy individuals, reactivation occurs frequently, mostly with asymptomatic or low, symptomatic viral shedding (7). However, clinically severe diseases due to EBV reactivation (such as allograft rejection and malignant B cell lymphoproliferation) can occur when host immunity is compromised during iatrogenic immunosuppression following solid organ transplant (SOT) or bone marrow transplant (BMT) (8).

The recognition of the clinical importance of CMV disease in the setting of immunodeficiency and immunosuppression, as well as the causative association of EBV concerning posttransplant lymphoproliferative disorders (PTLDs), has led to the implementation of clinical guidelines for screening, diagnosis, prevention, and treatment of CMV and EBV infections (9–12).

Nowadays, diagnostic procedures mainly rely on serological and molecular assays (13). Serology for the detection of CMV- and EBV-specific antibodies is the parameter of choice to identify primary infection. In the context of transplantation, pretransplant serology is the most recommended procedure for the assessment of the immune status and, consequently, to assess the donor/recipient risk category for posttransplant disease (11, 13). Quantitative real-time PCR (qRT-PCR) is the gold standard for the detection and quantification of viral DNAemia, which is crucial for the identification of patients who are at risk for the development of CMV- and EBV-associated complications in the posttransplant setting, as DNAemia indicates viral replication (11). Highly sensitive and accurate quantification of CMV and EBV DNAemia in blood by qPCR has consequently become the cornerstone for the surveillance and guidance of preemptive therapy strategies as well as antiviral prophylaxis and therapy monitoring (14, 15).

To provide standardized and interlaboratory comparable quantitative results, it is highly recommended that qPCR assays should be calibrated to WHO international reference standards and report viral loads in international units per milliliter (16–18). Nevertheless, transplant centers have already established long-standing specific clinical cutoff values (e.g., for the initiation of antiviral therapy) that rely on different reporting units. One of the most widespread units of reporting quantitative qPCR results used is copies per milliliter (19). If centers have implemented clinical cutoffs that rely on alternative reporting units other than international units per milliliter, local laboratories have to know if their results are comparable and interchangeable. Consequently, a reliable conversion (e.g., international units per milliliter to copies per milliliter) is needed to convert the results and provide comparable reports to the clinician.

In 2019, Qiagen introduced a new diagnostic platform, the NeuMoDx platform, into the market. The system is available in two sizes, either the NeuMoDx 96 system, with a maximum throughput of 144 samples within 8 h, or a larger version called the NeuMoDx 288 system, with a maximum throughput of 288 samples within 8 h. The system combines fully automatized nucleic acid extraction, amplification, and detection on a random-access platform with the ability to continuously load and perform different qualitative or quantitative PCR assays simultaneously. The platform-specific test kits available for the quantification of CMV and EBV DNA from EDTA plasma are standardized against the first WHO international standards and marketed as highly sensitive, with a turnaround time per analysis of about 60 min. These specifications could be of major advantage for laboratories managing increasing numbers of samples to be tested in the context of transplant management. Therefore, the primary aim of this single-center evaluation study, using WHO standards and archived clinical specimens, was to evaluate the analytical performance of the CE/IVD (*in vitro* diagnostic devices that adhere to the European In-Vitro Diagnostic Devices Directive [IVDD 98/79/EC])-certified NeuMoDx platform (NeuMoDx Molecular, a Qiagen Company, Ann Arbor, USA) for the detection and quantification of CMV and EBV DNA in EDTA plasma against the established reference routine qPCR assays. As no conversion from international units per milliliter to copies per milliliter was provided by the manufacturer, the second aim was to calculate and establish a conversion factor or formula for the output of results in copies per milliliter for CMV and EBV.

## RESULTS

### Analytical performance per WHO reference standards.

**FIG 1:**
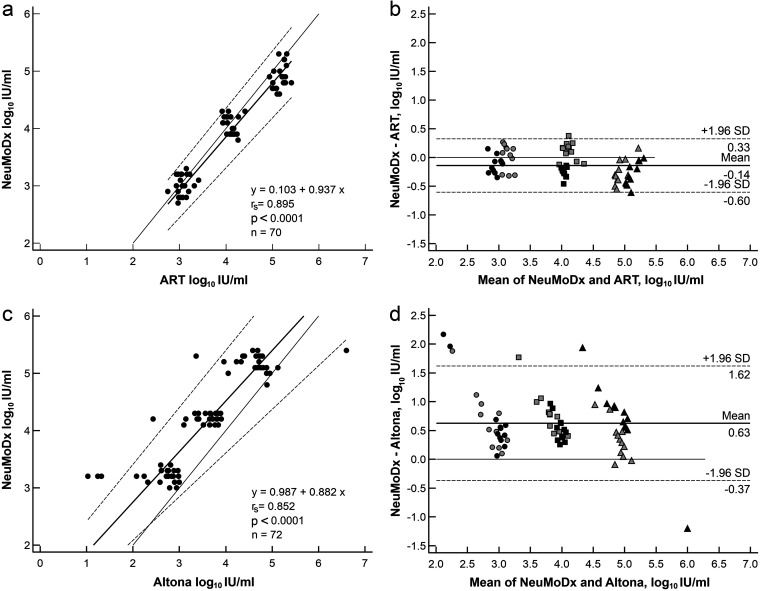
Scattergrams with Passing-Bablok regression and Bland-Altmann plots of serial dilutions of the 1st WHO international standards for CMV (a and b) and EBV (c and d).

Serial dilutions of the first WHO international standards for CMV and EBV, either in EDTA plasma or phosphate-buffered saline (PBS), with expected values of 5, 4, and 3 log_10_ IU/mL, were tested first in replicates of 12 in order to determine agreement, calculate correlation with the routine assays, and test for intra-assay variability between the NeuMoDx platform and the routine assays (Abbott RealTime m2000rt [ART] for CMV, Altona for EBV). Two CMV samples (2/72; 5 log_10_ IU/mL; one in PBS, one in EDTA plasma) were excluded because, after repeated testing due to unresolved results on the NeuMoDx platform, there was a lack of sample material for the Abbott platform. Results showed a qualitative agreement between the platforms of 100% (CMV, *n* = 70; EBV, *n* = 72) (Tables 1 and 2; Fig. 1). A low coefficient of variation (CV) was found in the intra-assay variability of the CMV tests on the NeuMoDx (CV range, 1.32% to 5.37%) and the ART platform (CV range, 1.15% to 4.38%). In contrast, high intra-assay variability was noted on the Altona workflow (CV range, 6.11% to 26.13%) compared to the NeuMoDx assay (CV range, 1.26% to 3.5%). Interassay variability was determined to range between 1.59% to 5.13% and 1.00% to 5.2% for the NeuMoDx CMV and EBV assays, respectively. Tested standard samples were strongly correlated with the respective comparison tests (CMV, Spearman correlation coefficient [*r_s_*] = 0.895, *P* < 0.001; EBV, *r_s_* = 0.852, *P* < 0.0001) and therefore in high agreement. The mean differences for CMV and EBV were −0.14 log_10_ IU/mL and 0.63 log_10_ IU/mL, respectively. Figure 1 presents scattergrams with Passing-Bablok regression and Bland-Altmann plots. No evidence of a systematic or proportional difference was found for CMV. For EBV, a systematic difference (intercept, 0.987; 95% confidence interval [CI], 0.561 to 1.495) and a proportional difference (slope, 0.882; 95% CI, 0.762 to 0.995) were detectable. No significant matrix effect (WHO reference standard in EDTA plasma versus PBS) was notable.

**TABLE 1:** Intra- and interassay variability as calculated from dilution series of 1st WHO standards for CMV

Dilution material (expected value [log_10_ IU/mL])	Intra-assay variability of:				Interassay variability of:	
	NeuMoDx (mean [SD])	NeuMoDx (CV [%])	ART (mean [SD])	ART (CV[%])	NeuMoDx (mean [SD])	NeuMoDx (CV[%])
EDTA (3.00)	3.13 (0.12)	3.69	3.27 (0.14)	4.38	3.1 (0.15)	4.82
EDTA (4.00)	4.18 (0.11)	2.73	4.20 (0.15)	3.48	4.08 (0.07)	1.84
EDTA (5.00)	4.86 (0.26)	5.37	5.22 (0.08)	1.53	5.25 (0.08)	1.59
PBS (3.00)	2.88 (0.11)	3.67	3.16 (0.11)	3.35	2.62 (0.07)	2.88
PBS (4.00)	3.91 (0.05)	1.32	4.32 (0.05)	1.20	3.87 (0.19)	5.13
PBS (5.00)	4.98 (0.18)	3.61	5.42 (0.06)	1.15	4.93 (0.10)	2.09

**TABLE 2:** Intra- and interassay variability as calculated from dilution series of 1st WHO standards for EBV

Dilution material (expected value [log_10_ IU/mL])	Intra-assay variability of:				Interassay variability of:	
	NeuMoDx (mean [SD])	NeuMoDx (CV [%])	Altona (mean [SD])	Altona (CV [%])	NeuMoDx (mean [SD])	NeuMoDx (CV [%])
EDTA (3.00)	3.18 (0.11)	3.50	2.52 (0.48)	18.93	3.15 (0.14)	4.38
EDTA (4.00)	4.20 (0.07)	1.76	3.39 (0.37)	11.07	4.18 (0.1)	2.35
EDTA (5.00)	5.07 (0.11)	2.12	4.73 (0.29)	6.11	5.17 (0.05)	1.00
PBS (3.00)	3.23 (0.10)	3.05	2.54 (0.66)	26.13	3.08 (0.16)	5.2
PBS (4.00)	4.23 (0.08)	1.84	3.71 (0.18)	4.76	4.28 (0.17)	4.02
PBS (5.00)	5.31 (0.07)	1.26	4.59 (0.75)	16.39	5.42 (0.07)	1.39

### Analytical performance in clinical specimens.

**(i) Overall concordance of clinical specimens.** After initial testing of the sample set on the NeuMoDx platform, 11 CMV (11/290) and 9 EBV (9/254) samples yielded invalid results, resulting in dropout quotes of 3.8% and 3.5%, respectively. Therefore, total numbers of 279 results for CMV and 245 results for EBV were available for comparative analysis. Analysis of primary testing results (see Table 3, step 1) showed an overall qualitative concordance of 91.0% (*n* = 25 discrepant) for CMV and 95.5% (*n* = 11 discrepant) for EBV. In addition, quantitative results obtained on the NeuMoDx platform were also analyzed to identify samples yielding a quantitative difference exceeding ±0.5 log_10_ IU/mL in comparison to the preexisting test results. Divergent quantitative results were noted in 25 (9.0%) samples for CMV and 19 (7.8%) for EBV (Table 3, step 1). Thus, the sum of discrepant results (quantitative and qualitative discrepancies) was 36 (12.9%) cases for CMV and 24 (9.8%) cases for EBV. Consequently, in order to exclude possible negative side effects of storage conditions on the sample material, all samples with discrepant qualitative results (see Table 3; discrepant results shown in bold) or divergent quantitative values of ±0.5 log_10_ IU were retested in a second step with the routine workflow for CMV or EBV.

**TABLE 3:** Overall concordance of CMV and EBV DNA measurements between the currently used assays ART (CMV) and Altona (EBV) and the respective NeuMoDx assays (CMV, *n* = 279; EBV, *n* = 245)^*a*^

Assay and characteristic	NeuMoDx result (no. [%])				
	TND	<LLOQ	Quantifiable	Total	ΔLog_10_ IU/mL > 0.5
ART CMV^*b*^					
TND	59 (21.1)	**1 (0.4)**	**2 (0.7)**	62 (22.2)	
<LLOQ	**13 (4.7)**	2 (0.7)	**4 (1.4)**	19 (6.8)	
Quantifiable	**9 (3.2)**	**7 (2.5)**	182 (65.2)	198 (71.0)	25 (9.0)
Total	81 (29.0)	10 (3.6)	188 (67.4)	279 (100.0)	
ART CMV^*c*^					
TND	72 (25.8)	0 (0.0)	0 (0.0)	72 (25.8)	
<LLOQ	**4 (1.4)**	4 (1.4)	**5 (1.8)**	13 (4.7)	
Quantifiable	**5 (1.8)**	**6 (2.2)**	183 (65.6)	194 (69.5)	7 (2.5)
Total	81 (29.0)	10 (3.6)	188 (67.4)	279 (100.0)	
Altona EBV^*d*^					
TND	42 (17.1)	**2 (0.8)**	0 (0.0)	44 (18.0)	
<LLOQ	**2 (0.8)**	5 (2.0)	0 (0.0)	7 (2.9)	
Quantifiable	**7 (2.9)**	**13 (5.3)**	174 (71.0)	194 (79.2)	19 (7.8)
Total	51 (20.8)	20 (8.2)	174 (71.0)	245 (100.0)	
Altona EBV^*e*^					
TND	44 (18.0)	**1 (0.4)**	0 (0.0)	45 (18.4)	
<LLOQ	**3 (1.2)**	7 (2.9)	0 (0.0)	10 (4.1)	
Quantifiable	**4 (1.6)**	**12 (4.9)**	174 (71.0)	190 (77.6)	3 (1.22)
Total	51 (20.8)	20 (8.2)	174 (71.0)	245 (100.0)	

a Discrepant results are shown in bold. <LLOQ, below the lower limit of quantification.

b Data represent concordance with the NeuMoDx CMV results (step 1).

c Data represent concordance with NeuMoDx CMV results with retested divergent samples (step 2).

d Data represent concordance with NeuMoDx EBV results (step 1).

e Data represent concordance with NeuMoDx EBV results with retested divergent samples (step 2).

After retesting the samples with discrepant results, overall qualitative concordance was 96.8% (270/279) for CMV and 96.7% (237/245) for EBV (see Table 3, step 2), indicating negative influences of storage conditions on some of the discrepant sample materials.

**FIG 2:**
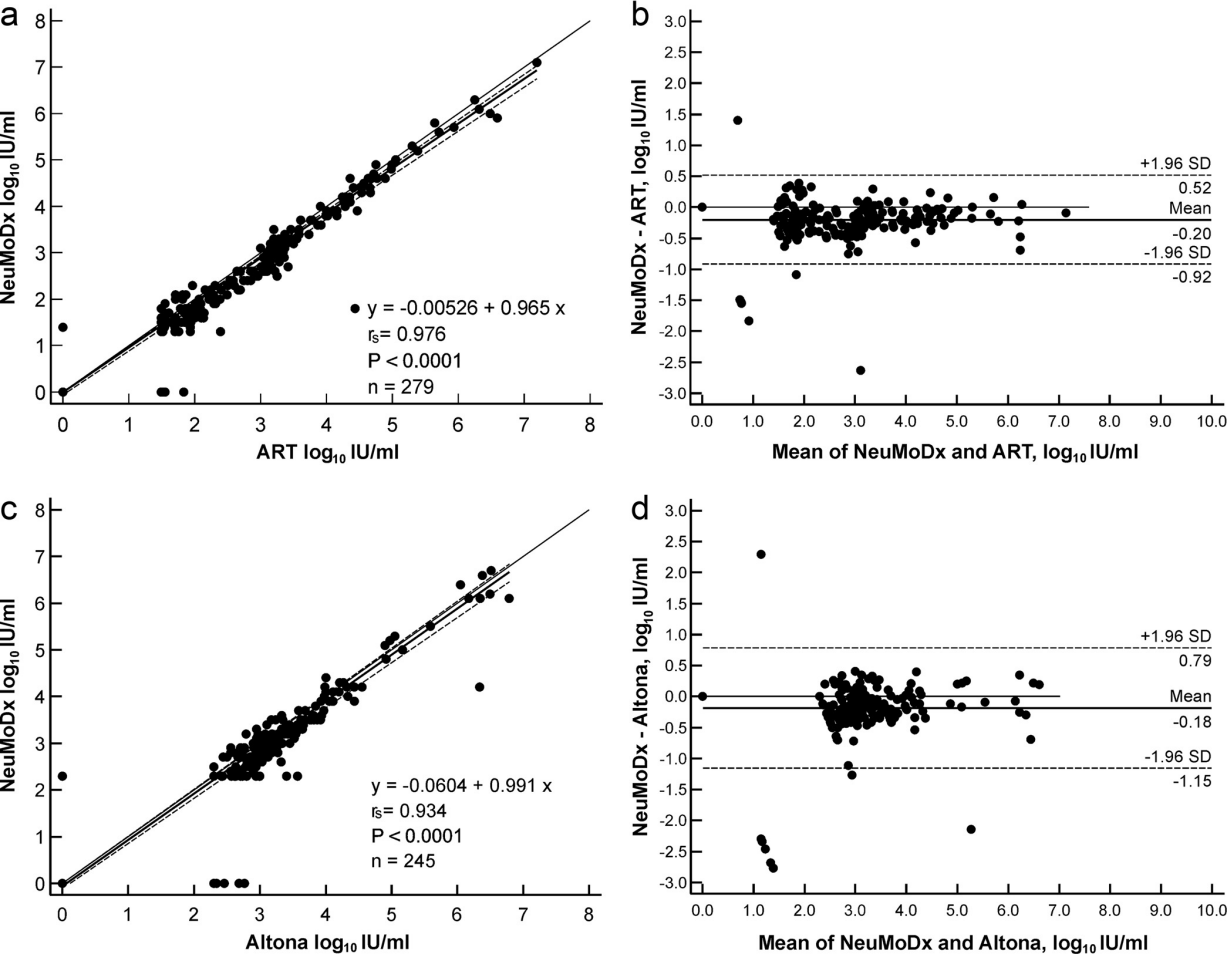
Scattergrams with Passing-Bablok regression and Bland-Altmann plots of leftover material from clinical samples for CMV (a and b) and EBV (c and d).

*(a) CMV*. The final CMV test results categorized by the limit of quantification of the assays (see Table 3, CMV, step 2) revealed that 4 samples were tested below the lower limit of quantification (LLOQ) by ART CMV but negative (target not detected [TND]) on the NeuMoDx platform. Five samples were quantifiable on the ART platform (ART mean, 1.59 log_10_ IU/mL; standard deviation [SD], 0.13) but negative (TND) on the NeuMoDx platform, whereas 6 samples were quantifiable on the ART system (mean, 1.8 log_10_ IU/mL; SD, 0.33) but less than the LLOQ on the NeuMoDx platform. In contrast, 5 samples had quantifiable results on the NeuMoDx platform (mean, 1.54 log_10_ IU/mL; SD, 0.18) but tested less than the LLOQ by ART. Seven samples (2.5%) with quantifiable results on both assays showed a quantitative difference of more than ±0.5 log_10_ IU/mL (ART mean, 3.10 log_10_ IU/mL; SD, 2.08; and NeuMoDx mean, 2.47 log_10_ IU/mL; SD, 1.59). The mean difference between both CMV tests was −0.2 log_10_ IU/mL (Fig. 2a and b). There was no evidence of a systematic difference (−0.005; 95% CI, −0.062 to 0.000), but a proportional difference (0.965; 95% CI, 0.947 to 0.978) was found between both CMV tests, with an *r_s_* of 0.976 (*P* < 0.001).

*(b) EBV*. After retesting the initially discrepant samples on the Altona EBV workflow (see Table 3, EBV, step 2), 3 samples (1.2%) were detected as below the LLOQ by Altona but negative (TND) on the NeuMoDx system, 4 samples (1.6%) were within the linear range of the Altona EBV assay (mean, 2.56 log_10_ IU/mL; SD, 0.2) but negative (TND) on the NeuMoDx platform, and 12 samples (4.9%) had quantifiable results (mean, 2.84 log_10_ IU/mL; SD, 0.36) but detected below the LLOQ on the NeuMoDx platform. One sample (0.4%) was detected below the LLOQ on the NeuMoDx but negative (TND) by the Altona EBV assay. Three samples (1.2%) with quantifiable results on both assays showed a quantitative difference of more than ±0.5 log_10_ IU/mL (Altona EBV, mean, 5.48 log_10_ IU/mL; SD, 1.89, versus NeuMoDx EBV, mean, 4.3 log_10_ IU/mL; SD, 1.75).

A strong correlation between both EBV tests (*r_s_* = 0.934, *P* < 0.001) with a mean difference of −0.18 log_10_ IU/mL (Fig. 2c and d) was observed without any evidence of a systematic (−0.060; 95% CI, −0.114 to 0.000) or proportional difference (0.991; 95% CI, 0.968 to 1.007).

### Conversion from log_10_ international units per milliliter to copies per milliliter.

Due to the high intra-assay variability noted for the Altona EBV assay in the WHO international standard dilutions and in order to minimize the risk of calculation bias by maximizing sample size, the calculation of the conversion from log_10_ international units per milliliter to copies per milliliter was based on data of clinical samples that were quantifiable in both assays. Tukey’s range test was used to identify and exclude significant outliers (CMV, *n* = 1; EBV, *n* = 4), resulting in a total of 182 samples for CMV and 170 samples for EBV (see Fig. S1 in the supplemental material).

For CMV, the fitted regression model was

$$\text{result in copies}/\text{mL}=10{\;}^{\left[0.0933\;+\;\left(0.\text{9722}\;\times \;{\text{result in log}}_{\text{1}0}\text{IU}/\text{mL}\right)\right]}$$

The overall regression was statistically significant (*R*^2^ = 0.9674, *P* < 0.001). It was found that the NeuMoDx log_10_ IU/mL significantly predicts Abbott log_10_ copies/mL, with β equal to 0.9722 (95% CI, 0.946 to 0.9985; *P* < 0.0001). Without the constant term, β changes to 0.9995 (95% CI, 0.9899 to 1.0090; *P* < 0.001), with a significant decrease in the data fitting (*P = *0.028, likelihood ratio test).

For EBV, the regression model was

$$\text{result in copies}/\text{mL}=10^{\;\left[0.3292\;+\;\left(0.\text{9375}\;\times \;{\text{result in log}}_{\text{1}0}\text{IU}/\text{mL}\right)\right]}$$

The overall regression was statistically significant (*R*^2^ = 0.935, *P* < 0.001). It was found that the NeuMoDx log_10_ IU/mL significantly predicts Altona log_10_ copies/mL, with β equal to 0.9375 (95% CI, 0.8999 to 0.9750; *P* < 0.001). Without the constant term, β changes to 1.0303 (95% CI, 1.0203 to 1.0403; *P* < 0.001), with a significant decrease in the data fitting (*P* < 0.001, likelihood ratio test).

### Time to result.

The mean time to result in April 2021 (ART for CMV, *n* = 1,445; Altona for EBV, *n* = 657) was 37 h for both CMV and EBV. The mean time to result in April 2022 (NeuMoDx for CMV, *n* = 1,482, and EBV, *n* = 792) was 14 h and 19 h for CMV and EBV, respectively.

## DISCUSSION

Quantification of CMV/EBV DNA in blood using real-time PCR assays is the currently accepted gold standard for surveillance and guidance of preemptive therapy strategies as well as antiviral prophylaxis and therapy monitoring in SOT and BMT patients (9, 11, 15). Although there is still a lack of consensus concerning the optimal sample type (whole blood versus EDTA plasma versus leukocytes) (20–24), most CMV and EBV assays are mainly validated for the quantification from plasma samples. Especially for hematopoietic stem cell transplantation, the independency of the total number of circulating white blood cells when performing analysis from plasma samples is the main advantage (24). Detection and quantification of very small amounts of viral DNA are prerequisites for clinical transplant management, as they allow an early assessment of viral load kinetics. Thus, clinical laboratories need sensitive and highly accurate standardized assays that ensure reproducible quantification of viral DNA. A maximum sample throughput with a short turnaround time, minimized hands-on time, and high flexibility is required in order to provide the results to clinicians as fast as possible (25). As the newly introduced NeuMoDx platform is able to process and combine multiple different CE/IVD-certified PCR tests independently with no need for batch processing and at short turnaround times, we evaluated the analytical performance of the quantitative CE/IVD-certified NeuMoDx CMV and EBV Quant assays on the NeuMoDx 96 platform in clinical specimens and WHO reference standard samples. We compared the whole workflow to our established routine tests (Abbott RealTime CMV kit and the Altona RealStar EBV test) with the intention to change to a faster and more flexible, but still highly standardized, workflow meeting the requirements of current quality assurance.

We found a high overall concordance and excellent correlation of viral load assessment in clinical specimens with the previous routine assays for CMV (96.7%; 270/279; *r_s_* = 0.976, *P* < 0.0001) and EBV (96.7%; 237/245; *r_s_* = 0.934, *P* < 0.0001). Replicate testing of WHO reference samples for CMV and EBV showed that intra-assay variability was low between the two automated platforms (NeuMoDx and ART) but was remarkably high for the Altona EBV qPCR workflow. As this assay was already standardized against the 1st WHO international standard, and we did not find a significant matrix effect on the automated platforms, the high deviation of the EBV test may mainly be due to manual DNA extraction prior to qPCR analysis. This assumption is in concordance with previously published literature concerning workflow automatization (25–27). Consequently, the high intra-assay variability of the Altona EBV assay seen in the WHO replicate testing was the reason to calculate the conversion formula from data of clinical samples. The strategy of maximizing sample size in order to minimize the risk of calculation bias has led to the calculation of a reliable conversion formula. Due to an improvement in the data fitting (according to the likelihood ratio test), we decided to implement the conversion formula, rather than the conversion factor (without a constant).

To our knowledge, this is one of the first studies evaluating the analytical and clinical performance of the CMV and EBV qPCR assays on the NeuMoDx platform and the first study to calculate a conversion formula from log_10_ international units per milliliter to copies per milliliter. The strength of this study lies in the comparison of both assays under identical laboratory settings using WHO reference standard materials as well as a high number of clinical specimens covering the most critical medical decision ranges.

In concordance with both Luciani et al. (28), who evaluated the NeuMoDx platform and assay for CMV, and Mourik and colleagues (29), who evaluated NeuMoDx for both CMV and EBV, qualitative agreement with previously established tests was good. However, while Luciani et al. (28) found the NeuMoDx CMV assay too sensitive clinically, the sensitivity is in excellent concordance with our previous routine test (Abbott m2000 and RealTime CMV assay). In contrast to Mourik and colleagues (29), we found a good quantitative agreement for both NeuMoDx assays. The difference might be accounted for by our higher numbers of samples quantifiable in both NeuMoDx and the respective reference test (30). The most relevant limitation of this study is the use of archived clinical specimens. Samples had been stored for up to 5 years before the study. Although sample storage conditions were in concordance with established guidelines (31), a negative influence could not be ruled out. Therefore, retesting of the samples in question was performed on the respective reference test system. A similar sample storage influence was noted in the study by Mourik et al. (29). Furthermore, the inclusion of follow-up samples for the comparison of assay stability and comparability was impossible because of a lack of sufficient stored leftover samples.

In implementing the MeuMoDx platform in routine clinical laboratory practice, we found that time to result improved, especially due to the possibility of continuous loading. This is also reported by Luciani et al. (28).

In conclusion, we validated the analytical performance and evaluated the comparative clinical performance, as well as a conversion formula, to report results in copies per milliliter, using the NeuMoDx 96 platform to detect and quantify CMV and EBV DNA in EDTA plasma specimens. We have found a high overall concordance and a significant correlation of quantitative values for both assays. Comparing the automated workflow on the NeuMoDx platform to the manual workflow for the detection of EBV, the study results emphasize the advantage of fully automated workflows. As the system enables highly accurate and rapid detection of CMV and EBV, we consequently introduced the NeuMoDx platform to our routine analysis for the quantitative detection of CMV and EBV in EDTA plasma samples in December 2021, and since then, we have applied our calculated conversion factor to report NeuMoDx CMV and EBV results in copies per milliliter. This allowed us to significantly shorten our turnaround times and therefore provide most results even on the same day to the clinicians.

## MATERIALS AND METHODS

### Specimens.

**(i) International reference standards.** The first WHO international standards (WHO-IS) for CMV and EBV (32, 33) were used for the preparation of dilution series for multiple-replicate testing. Both WHO international standards had an expected concentration of 5 × 10^6^ IU/mL (i.e., 6.7 log_10_ IU/mL) after reconstitution in 1 mL of water. Serial dilutions of the WHO international standards in PBS and EDTA plasma were prepared with expected values of 5, 4, and 3 log_10_ IU/mL. EDTA plasma for the dilutions was used from one anonymous donor with known seronegativity for CMV and EBV who additionally tested negative for active CMV and EBV DNA replication by routine reference qPCR assays. A total of 144 aliquots (72 for CMV and 72 for EBV) of dilutions in PBS (CMV, *n* = 36; EBV, *n* = 36) and EDTA plasma (CMV, *n* = 36; EBV, *n* = 36) were prepared (Table 4). To evaluate detection rate, quantitative agreement, linearity, and intra-assay variability, each dilution was separated into 12 aliquots, and all replicates were tested in a single run on the NeuMoDx platform and the respective reference routine assays. Two replicates of each dilution step were tested on 3 consecutive days on the NeuMoDx platform in order to determine the interassay variability of this assay.

**TABLE 4:** Specimen characteristics

Characteristic	Data for:	
	CMV (*n* = 290)	EBV (*n* = 254)
Clinical samples		
Total no. of patients	228	193
Age (years [median, IQR])	57 (15)	51 (28)
No. (%) male	144 (63.2)	116 (60.1)
No. (%) female	84 (36.8)	77 (39.9)
No. (%) of follow-up patients	39 (17.1)	31 (16.1)
No. (%) of follow-up samples	62 (21.4)	61 (24.0)
No. (%) TND	66 (22.8)	47 (16.2)
Below LLOQ (no. [%])	21 (7.2)	7 (2.4)
1.50–1.99 log_10_ IU/mL	39 (13.4)	NA^*a*^
2.00–2.99 or 2.31–2.99 log_10_ IU/mL^*b*^	43 (14.8)	64 (22.1)
3.00–3.99 log_10_ IU/mL	72 (24.8)	106 (36.6)
4.00–4.99 log_10_ IU/mL	37 (12.8)	19 (6.6)
5.00–5.99 log_10_ IU/mL	6 (2.1)	2 (0.7)
6.00–6.99 log_10_ IU/mL	5 (1.7)	9 (3.1)
7.00–8.00 log_10_ IU/mL	1 (0.3)	0 (0.0)
WHO standard dilution series (72 aliquots each)		
EDTA/PBS expected value (per log_10_ IU/mL)		
3.00	12/12	12/12
4.00	12/12	12/12
5.00	12^*c*^/12^*c*^	12/12

*^a^*The range of 1.50 to 1.99 is below the LLOQ for EBV.

*^b^*The range is 2.00 to 2.99 for CMV, and 2.31 to 2.99 for EBV (as the limit of quantification is 2.30).

*^c^*Only 11 of the 12 aliquots were tested on the ART because insufficient material was left after retesting on the NeuMoDx.

**(ii) Clinical EDTA plasma samples.** A computer-based query from the laboratory interface system was performed to identify applicable archived leftover EDTA plasma samples that have been tested by qPCR for CMV between February 2015 and January 2020 or for EBV between April 2015 and May 2020. Samples were selected to cover the whole linear range of the qPCR assays, with an emphasis on clinically and therapeutically the most relevant viral load between 2 and 4 log_10_ IU/mL. All archived samples used for this study have been stored at below −20°C within 24 h after initial routine qPCR analysis for CMV/EBV and have not undergone repetitive freeze/thaw cycles according to ISO 20186-3:2019 (31). Only samples with at least 1,500 μL of leftover sample material available were selected for testing and pseudonymized by assigning an internal identification number (ID). As this was a study on archived leftover clinical samples, original routine qPCR test results for CMV and EBV were already available for comparison, and retesting on routine assays was only performed in case of discrepant results. Discrepancy was *a priori* defined as either a qualitative difference (target not detected [TND] versus below limit of quantification [<LLOQ] or quantifiable) or, in the case of quantitative results on both platforms, a difference of more than 0.5 log_10_ IU/mL compared to the initial result.

In total, 544 archived EDTA plasma samples from 421 patients (CMV, *n* = 228; EBV, *n* = 193) with preexisting test results from initial routine testing were available for retesting on the NeuMoDx platform for this comparative study. Of these, 290 samples were tested for CMV and 254 for EBV. The selection comprised samples with preexisting negative results from routine testing (CMV, *n* = 66 [22.8%]; EBV, *n* = 47 [16.2%]), as well as quantitative values covering the linear range of the NeuMoDx platform (CMV, 1.3 to 7.1 log_10_ IU/mL; EBV, 2.3 to 6.7 log_10_ IU/mL), with an emphasis on the clinical and therapeutical most relevant viral load between 2 and 4 log_10_ IU/mL. All sample characteristics are summarized in Table 4.

### Ethical statement.

The study was approved by the local ethics committee of the Medical University of Vienna (EK no. 1757/2020).

### Instruments.

The specific sensitivities of the four different assays as indicated by the vendors, as well as the terms used to report CMV/EBV DNA viral loads in the present study, are summarized in Table S1 in the supplemental material. All tests were performed according to the instructions of the manufacturers.

### (i) Abbott m2000 and RealTime CMV assay.

The routine DNA extraction and amplification for CMV DNA quantification in EDTA plasma (500 μL) were performed using the Abbott RealTime CMV kit (CE/IVD; Abbott Molecular Inc., Des Plaines, IL) on the m2000 RealTime platform. DNA was extracted automatically on the m2000sp system with the Abbott mSample preparation system DNA. Quantitative real-time PCR was performed using the Abbott RealTime CMV amplification reagent kit on the Abbott RealTime m2000rt instrument. In the following text, this workflow is abbreviated as ART.

### (ii) LightCycler 480 II, Roche high pure extraction, and Altona RealStar EBV assay.

For EBV DNA quantification, the routine CE/IVD workflow was performed by manual DNA extraction from 200 μL of EDTA plasma, using the Roche high pure viral nucleic acid kit (Roche Diagnostics AG, Rotkreuz, Switzerland), followed by quantitative real-time PCR with the Altona RealStar EBV PCR kit 1.0, Epstein-Barr virus quantitative (Altona Diagnostics GmbH, Hamburg, Germany) on the Roche LightCycler 480 II (Roche Diagnostics AG). This workflow is abbreviated as Altona.

### (iii) NeuMoDx 96 platform.

For this comparative study, the fully automated, CE/IVD-certified NeuMoDx 96 platform (NeuMoDx Molecular, Inc., a Qiagen company, Ann Arbor, MI, USA) was used for the quantification of CMV and EBV DNA. The system was chosen for potential implementation at our lab because it can run several different RT-PCR assays at once and allows continuous random access with the ability to prioritize samples (short turnaround time samples [STAT]). The NeuMoDx 96 platform comprises room temperature reagent storage, nucleic acid extraction (based on magnetic beads), RT-PCR setup, amplification, and detection, as well as results analysis and reporting. For the NeuMoDx CE/IVD CMV Quant assay (NeuMoDx Molecular, Inc.) 550 μL or 250 μL of sample material for the NeuMoDx CE/IVD EBV Quant assay (NeuMoDx Molecular, Inc.) was used for each qPCR run. This Workflow is abbreviated as NeuMoDx.

### Time to result.

The division of clinical virology sample-handling IT system was queried for the time points of sample arrival at the lab and final result validation of all CMV and EBV tests in the months of April 2021 (ART for CMV, *n* = 1,445; Altona for EBV, *n* = 657) and April 2022 (NeuMoDx for CMV, *n* = 1,482, and EBV, *n* = 792). The mean time to result was calculated as the arithmetic mean of hours from sample arrival to final result.

### Statistical analyses.

Statistical analyses were performed using MedCalc 20.105 (MedCalc Software, Ostend, Belgium) and R (version 4.2; Vienna, Austria). The distribution of numerical data was assessed using the Kolmogorov-Smirnov test (with Lilliefors significance correction) and probability-probability plots (pp-plots). Numerical data were presented as mean ± standard deviation. The Spearman’s correlation coefficient (*r_s_*) and Passing-Bablok (PB) regression were used for method comparison (34). In these nonparametric linear regression models, no evidence of a systematic difference between the two methods is found when the 95% confidence interval (CI) of the intercept (*b*_0_) includes the value zero. Furthermore, a proportional difference can be precluded when the 95% CI of the slope (*b*_1_) is around the value of 1. As a graphical representation of the comparison results, Bland-Altman scatterplots (35) were applied. Before estimating a conversion factor, the Tukey outlier test was applied, and extreme outliers were excluded. Ordinary least-squares regression was used to calculate the conversion factor. To obtain the CMV or EBV viral load result in copies/mL, calculate the corresponding, *y* = 10^[constant term^^+^^(conversion factor^^×^*^x^*^)]^, where *x* is the initial result in log_10_ international units per milliliter, and *y* is the result in log_10_ copies per milliliter.

For simplicity, a linear regression model without constant terms was additionally applied. The likelihood ratio test of nested models (implemented in the lmtest R package) was used for the comparison between models with and without the constant term. Statistical significance was defined as *P* values of less than 0.05. Where appropriate, accumulation of alpha errors associated with multiple testing was controlled for by applying the Bonferroni-Holm method.
