# Supplementary material for: Performance Evaluation of the Fully Automated NeuMoDx RT-PCR Platform for the Quantification of CMV and EBV DNA in EDTA Plasma: Implications for Clinical Management and Establishment of a Conversion Formula
Source: Microbiol Spectr. 2022 Nov 7;10(6):e02157-22. doi: 10.1128/spectrum.02157-22 (PMC9769568; doi:10.1128/spectrum.02157-22)
Supplement: Supplemental file 1 — Supplemental material. Download spectrum.02157-22-s0001.pdf, PDF file, 0.5 MB [file spectrum.02157-22-s0001.pdf]

Supplementary Material

Performance evaluation of the fully-automated NeuMoDx RT-PCR platform for the quantification of CMV and EBV DNA in EDTA plasma: implications for clinical management and establishment of a conversion formula.

Authors: Anna Nele Herdina[a], Franz Ratzinger[b], Monika Breuer[a], Julia Schellnegger[a], Rui Qiang Chen[a], Thomas Watkins-Riedel[a], Nicole Perkmann-Nagele[a], Robert Strassl[a]#.

[a]Division of Clinical Virology, Department of Laboratory Medicine, Medical University of Vienna, Vienna, Austria
[b] IhrLabor, Medical Diagnostic Laboratories, Vienna, Austria
#Address correspondence to Robert Strassl, robert.strassl@meduniwien.ac.at.

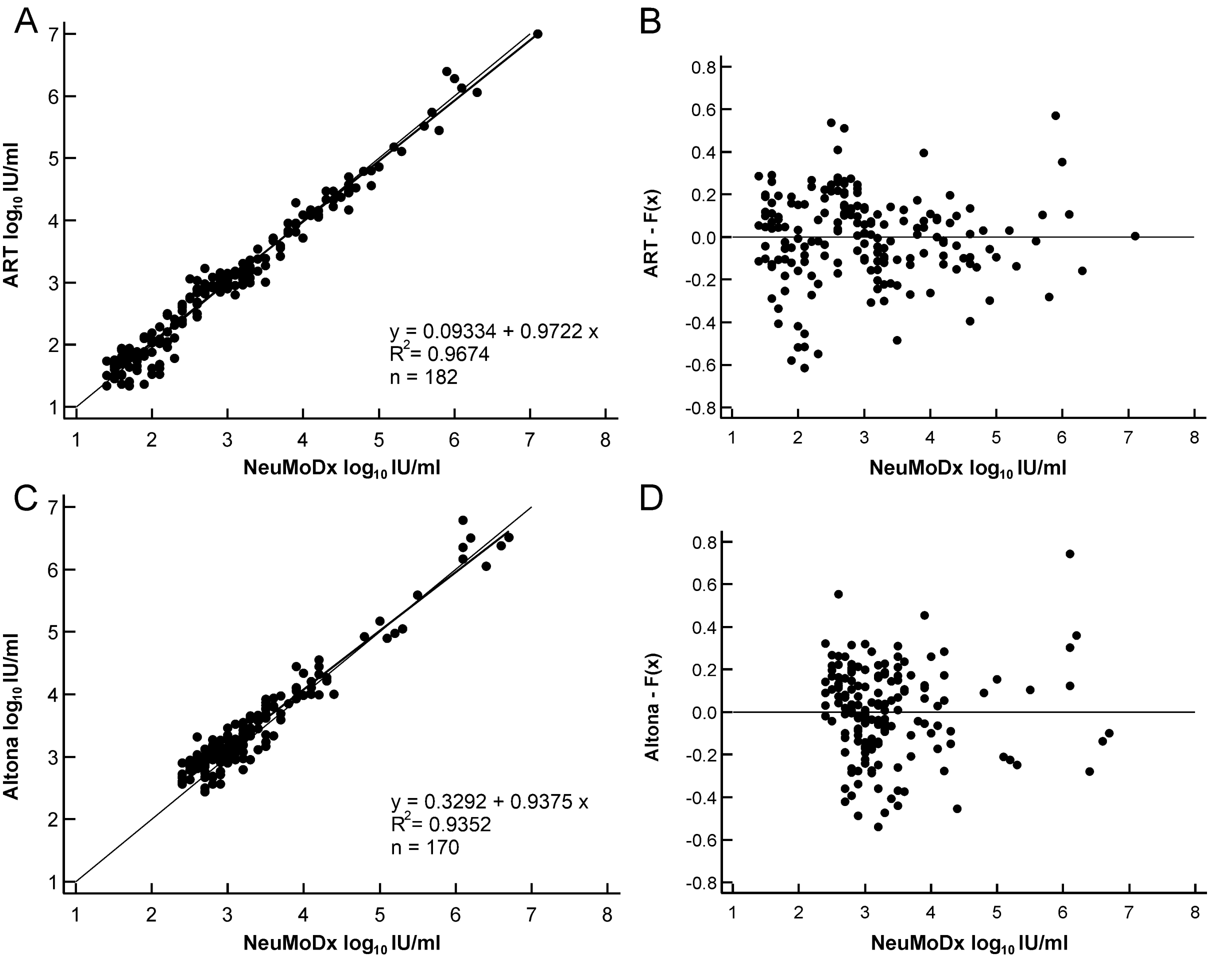

Supplementary Fig. S1: Conversion formula regression line and residuals for CMV (a/b) and EBV (c/d).

Supplementary Table S1 Assay specific sensitivities as indicated by the vendor and terms used to report CMV / EBV DNA results

	Assay			
	ART CMV	*NeuMoDx CMV Quant Assay*	*Altona EBV*	*NeuMoDx EBV Quant Assay*
Standardized against	1st WHO Standard 09/162	1st WHO Standard 09/162	1st WHO Standard 09/260	1st WHO Standard 09/260
Virus specific amplification targets	UL34 & UL80.5	UL54 & UL71	not specified	BALF5 & BXFL1
Lower limit of detection (LLOD)	31.2 IU/ml<br>1.49 log IU/ml<br>20 copies/ml plasma sample<br>[62.4 IU/ml, 1.80 log IU/ml, 40 copies/ml whole blood]	1.3 log IU/ml	1.1 copies/µl (in eluate)	2.3 log IU/ml
Lower limit of quantification (LLOQ)	31.2 IU/ml<br>1.49 log IU/ml<br>20 copies/ml plasma sample<br>[62.4 IU/ml, 1.80 log IU/ml, 40 copies/ml whole blood]	1.3 log IU/ml	2.3 log IU/ml<br>200 copies/ml	2.3 log IU/ml
Upper limit of quantification (ULOQ)	156 million IU/ml<br>8.19 log IU/ml<br>8.00 log copies/ml	8 log IU/ml	1E+07 copies/µl	8 log IU/ml
Linear range (for EDTA-plasma)	31.2 IU/ml to 156 million IU/ml<br>1.49 to 8.19 log IU/ml<br>20 copies/ml to 8.00 log copies/ml	1.3 - 8 log IU/ml	1E+01 to 1E+07 copies/µl<br>at least 7 orders of magnitude	2.3 - 8 log IU/ml
Validated sample materials	EDTA-plasma, whole blood (EDTA)	EDTA-plasma	EDTA-plasma, whole blood, cerebrospinal fluid	EDTA-plasma
Conversion factor (IU/ml to c/ml)	Manufacturer provided:<br>IU/ml = copies/ml x 1.56<br>log IU/ml = log copies/ml + 0.19	Calculated in the present study:<br>copies/ml=10^(0,09334+(0,9722 x logIU/ml))	Manufacturer provided:<br>IU/ml = copies/ml	Calculated in the present study:<br>copies/ml=10^(0,3292+(0,9375 x logIU/ml))
Workflow	semi-automated (manual transfer of prepared PCR-plates necessary)	fully automated	manual nucleic acid extraction, mastermix creation and PCR plate setup	fully automated
Sample process control (internal control)	Yes: vendor specific internal control	Yes: vendor specific internal control	Yes: vendor specific internal control	Yes: vendor specific internal control
Preparation hands-on time	30 min / 48 samples	20 min / day	1h 30 min / 24 samples	20 min / day
Result evaluation and transcription hands-on time	1 h / 48 samples	1 min / sample	40 min / 24 samples	1 min / sample
Total technical hands-on time	1 h 30 min / 48 samples	<2 min / sample	2 h 10 min / 24 samples	<2 min / sample
Time-to-result	24 samples: 5 h 28 min<br>48 samples: 6 h 32 min	DNA: 60 min / sample<br>RNA: 90 min / sample	24 samples: 3 h 45 min	DNA: 60 min / sample<br>RNA: 90 min / sample
Sample volume used	500 µl	550 µl	200 µl	250 µl
Minimal volume input (according to manual)	min. 600 µl (depending on tube size)	min. 650 µl (depending on tube size)	250 µl	min. 350 µl (depending on tube size)
Processing	batch (24 / 48 / 72 / 94)	continous loading	batch (flexible)	continous loading
Special characteristics		Sample priorization (STAT)		Sample priorization (STAT)
Audit trail	Yes	Yes	No (manually)	Yes

Nomenclature	***Interpretation***
TND: Target not detected	CMV DNA or EBV DNA not detected
TD: Target detected	CMV DNA or EBV DNA detected (either <LLOQ or quantifiable)
<LLOQ: Below the lower limit of quantification	CMV DNA or EBV DNA detected but <LLOQ
Exact CMV / EBV DNA concentration (IU/ml)	Quantifiable CMV DNA or EBV DNA concentration within the linear range of the assay
> ULOQ: Above the upper limit of quantification	CMV DNA or EBV DNA detected but >ULOQ

Supplementary Table S2: Conversion of results with the conversion formula or factor

To get the viral load result in copies/ml, calculate the respective CMV or EBV formula as

y = constant + (conversion factor * x)

where x is the initial result in logIU/ml and y is the result in log copies/ml.

Then calculate antilog for y (=10^y).

Alternatively, a simpler conversion without a constant can be applied as

y = conversion factor * x

Then antilog for y (=10^y) is calculated.

**with constant**	**without constant**
**CMV**	
constant (y-axis intercept) 0.0933	
conversion factor (β) 0.9722	conversion factor (β) 0.9995
e.g. for an initial CMV NeuMoDx result of 3.0 logIU/ml:	
1023 copies/ml=10^ [0.0933+(0.9722*3.0 logIU/ml)]	997 copies/ml=10^ (0.9995*3.0 logIU/ml)
**EBV**	
constant (y-axis intercept) 0.3292	
conversion factor (β) 0.9375	conversion factor (β) 1.0303
e.g. for an initial EBV NeuMoDx result of 3.0 logIU/ml:	
1386 copies/ml=10^ [0.3292+(0.9375*3.0 logIU/ml)]	1233 copies/ml=10^ (1.0303*3.0 logIU/ml)
